# Effects of zinc oxide and condensed tannins on the growth performance and intestinal health of weaned piglets in ETEC-challenged environment

**DOI:** 10.3389/fmicb.2023.1181519

**Published:** 2023-04-27

**Authors:** Hongbo Yi, Zhikang Wang, Bijing Yang, Xuefeng Yang, Kaiguo Gao, Yunxia Xiong, Qiwen Wu, Yueqin Qiu, Shenglan Hu, Li Wang, Zongyong Jiang

**Affiliations:** State Key Laboratory of Livestock and Poultry Breeding, Ministry of Agriculture Key Laboratory of Animal Nutrition and Feed Science in South China, Guangdong Key Laboratory of Animal Breeding and Nutrition, Maoming Branch, Guangdong Laboratory for Lingnan Modern Agriculture, Institute of Animal Science, Guangdong Academy of Agricultural Sciences, Guangzhou, China

**Keywords:** zinc oxide, condensed tannins, intestinal health, *Escherichia coli*, weaned pig

## Abstract

This experiment was conducted to evaluate effects of zine oxide (ZnO) and condensed tannins (CT), independently or in combination, on the growth performance and intestinal health of weaned piglets in enterotoxigenic *Escherichia coli* (ETEC-K88)-challenged environment. Randomly divided 72 weaned piglets into 4 groups. Dietary treatments included the following: basic diet group (CON), 1,500 mg/kg zinc oxide group (ZnO), 1,000 mg/kg condensed tannins group (CT), and 1,500 mg/kg zinc oxide +1,000 mg/kg condensed tannins group (ZnO + CT). Dietary ZnO supplementation decreased diarrhea rate from 0 to 14 days, 15 to 28 days, and 0 to 28 days (*p* < 0.05) and no significant on growth performance. The effect of CT on reducing diarrhea rate and diarrhea index was similar to the results of ZnO. Compared with the CON group, ZnO increased the ileum villus height and improved intestinal barrier function by increasing the content of mucin 2 (MUC-2) in jejunum and ileum mucosa and the mRNA expression of zonula occludens-1 (ZO-1) in jejunum (*p* < 0.05) and the expression of Occludin in duodenum and ileum (*p* < 0.05). The effects of CT on intestinal barrier function genes were similar to that of ZnO. Moreover, the mRNA expression of cystic fibrosis transmembrane conductance regulator (CFTR) in jejunum and ileum was reduced in ZnO group (*p* < 0.05). And CT was also capable of alleviating diarrhea by decreasing CFTR expression and promote water reabsorption by increasing AQP3 expression (*p* < 0.05). In addition, pigs receiving ZnO diet had higher abundance of phylum *Bacteroidetes*, and genera *Prevotella*, and lower phylum *Firmicutes* and genera *Lactobacillus* in colonic contents. These results indicated that ZnO and CT can alleviate diarrhea and improve intestinal barrier function of weaned pigs in ETEC-challenged environment. In addition, the application of ZnO combined with CT did not show synergistic effects on piglet intestinal health and overall performance. This study provides a theoretical basis for the application of ZnO in weaning piglet production practices, we also explored effects of CT on the growth performance and intestinal health of weaned piglets in ETEC-challenged environment.

## 1. Introduction

Piglets are susceptible to a series of effects such as nutrition, pathogenic microorganisms, and environment after weaning, resulting in reduced feed intake, stunted growth performance, and diarrhea (Hu et al., 2018). Previous studies have shown that zinc oxide (ZnO) supplementation to the diet of weaned piglets enhanced growth performance, improves intestinal morphology (Conway et al., 2022), and has a positive effect on immune function (Pei et al., 2019). At present, ZnO plays a crucial role in the prevention of diarrhea in weaned piglets. It has been reported that the maximum allowable supplemental dose of ZnO within 14 days after weaning is 2,500 mg/kg in most parts of the world (Nielsen et al., 2021). However, soil and water pollution caused by the use of high doses of ZnO has become a serious problem.

Tannins are the largest polyphenols in plant extracts and can be classified into hydrolysable or condensed subgroups (Caprarulo et al., 2021). In recent years, it has been found that extracted tannins have been widely exploited on intensive pig farms and used in different physiological stages of pigs (especially after weaning; Girard et al., 2020; Girard and Bee, 2020). Starčević et al. found that condensed tannins (CT) have a significant effect on improving animal performance, antibacterial activity and regulating intestinal flora (Starčević et al., 2015). Several other studies have reported that CT also enhance the antioxidant capacity, regulate intestinal microbiome composition, and improve the quality of the meat (Reggi et al., 2020; Buyse et al., 2021). This shows the great potential of CT feedstuff development and utilization.

To our knowledge, there is limited data about the effects of dietary supplementation with ZnO and CT on growth performance, antioxidation, and intestinal health of weaned pigs. In the K88-polluted environment in this study, we added CT to the diets of weaned piglets and combined it with ZnO to explore whether CT could substitute for ZnO, and whether the combination of ZnO and CT would have interactive effects on growth performance and intestinal health.

## 2. Materials and methods

All animal experimental protocols used in present study were according to the Chinese guidelines for animal welfare and approved by the Animal Care and Use Committee of Guangdong Academy of Agricultural Sciences.

### 2.1. Reagents

ZnO is feed grade (Guangzhou Zelong Chemical Co, China). CT were extracted from Kenwood, with molecular mass of 1700-1900u, consisting of 33% dimer, 37% trimer, 21% tetramer, 8% pentamer, and 1% heptamer (Guangzhou Youbei Technology Co, China). The hand-held sprayer for spraying mushrooms is produced by Guangzhou Yitai Zheng Co, China.

### 2.2. *Escherichia coli* liquid preparation

The enterotoxigenic *Escherichia coli* (ETEC-K88) used in this experiment was purchased from China Veterinary Drug Control Institute. The strain was thawed from the refrigerator at −20°C, added into the centrifugation tube with 10 ml Luria-Bertani (LB) broth culture medium, and the tubes were cultured in an incubator at 37°C for 12 h. Thereafter, the 10 ml LB broth was poured into a triangular flask containing 2 l LB broth, and cultured at 37°C for 18 h. The LB broth was stored in at 4°C refrigerator and used as a concentrated bacterial solution.

### 2.3. Animal treatment

A total of 72 healthy (*Duroc* × *Landrace* × *Yorkshire*, 26 ± 2 days of age, body weight of 8.40 ± 0.20 kg) weaned piglets were divided into 4 groups according to the principle of similar weight with half male and half female in each group, 6 replicates per group, 3 pigs per replicate, and the diet treatment was divided into basic diet group (CON), 1,500 mg/kg zinc oxide group (ZnO), 1,000 mg/kg condensed tannins group (CT), and 1,500 mg/kg zinc oxide +1,000 mg/kg condensed tannins group (ZnO + CT). The basal diet (Supplementary Table S1) was formulated according to NRC to meet the nutritional requirements of piglets from 7 to 11 kg. During the whole experiment, the animals were free to access feed and water. The piggery should not be disinfected before the start of the test. After the start of the experiment, the *Escherichia coli* liquid should be sprayed every 4 days without flushing the piggery. The specific method is as follows.

### 2.4. Simulation of K88 challenged environment

The 200 ml of concentrated bacterial solution was diluted with saline to 3 L as the working bacterial solution, and the working bacterial solution was poured into the electric sprayer (the flow rate was 250 ml/min), and the nozzle was aligned with the partition, the slatted floor, the drinking fountain, and the feeding trough. After spraying, the concentration of working bacterial solution was collected from the nozzle of the spray machine for verification. After gradient dilution, the bacterial liquid was dropped onto the eosin methylene blue medium for 24 h. The colonies were counted and the concentration of the working bacterial liquid was calculated to be 6 × 10^8^ CFU/ml. The bacteria were sprayed once on the first day of the experiment, and every 4 days thereafter, for a total of 7 times (the 1st, 5th, 9th, 13th, 17th, 21st, and 25th days of the experiment).

### 2.5. Sample collection

The experimental period was 28 days. On the morning of the 15th day of the trial, one pig from per pen was weighed and blood was collected from the anterior vena cava. Then, plasma and serum were separated stored at −80°C. After the pigs sacrificed, and intestinal segments of approximately 2 cm of proximal duodenum, middle jejunum, and distal ileum were selected then washed with phosphate buffered saline (PBS) and placed in 4% paraformaldehyde for overnight fixation. The liver, proximal duodenum, mid-jejunum, distal ileum, and intestinal mucosa were collected and quickly placed in liquid nitrogen, and then stored at −80°C for long-term storage. The colonic contents were collected and put into liquid nitrogen for the detection of intestinal microorganisms.

### 2.6. Measurement of serum and liver antioxidant capacity

The activities of total antioxidant capacity (T-AOC, A015-2-1), malondialdehyde (MDA, A003-2), total superoxide dismutase (T-SOD, A001-1), and glutathione peroxidase (GSH-Px, A005) were determined by commercial kits provided by Nanjing Jiancheng Institute of Bioengineering (Nanjing, China). The liver samples were homogenized with normal saline and centrifuged to obtain supernatant. Then, the supernatant was diluted to 10% for determination. The protein concentrations of sample were measured using a bicinchoninic acid assay (BCA) kit (Thermo Fisher, USA, 23225), and results were expressed as per milligram protein.

### 2.7. Enzyme-linked immunosorbent assay

The concentration of inflammation cytokines (IL-1B, ml025973, TNF-α,ml002360 TGF-β, ml002363, IL-6, ml025981 IL-8, ml02598, IL-10, and ml025956) and immunoglobulin (IgM, ml002334, IgG, and ml002328,) in serum was determined by ELISA kits (Shanghai Meilian Biotechnology Co., Ltd., Shanghai, China), as well as the concentration of secreted immunoglobulin (sIgA, ml026686) and mucin-2 (MUC-2) in jejunum and ileum mucosa. The mucosa of jejunum and ileum was added into normal saline to make 10% homogenate, and supernatant was collected after centrifugation at low temperature for 10 min, finally detected them according to the instructions.

### 2.8. The intestinal morphology examination

Briefly, the fixed duodenum, jejunum, and ileum were dehydrated in a gradient manner, then embedded in paraffin, cut into 5 μm thick sections, and stained with hematoxylin and eosin (HE). Finally, images of intestinal morphology were captured using camera fitted light microscope. Pannoramic Viewer was used to measure intestinal villus height and crypt depth, and 5 fields were randomly selected for observation and measurement in each slice.

### 2.9. Quantitative real-time polymerase chain reaction

Total RNA was extracted from the jejunum, ileum and colon using Trizol reagent (Invitrogen, Carlsbad, CA, USA) according to manufacturer instructions. The purity of RNA was confirmed by measuring absorbance at 260 nm and 280 nm using NanoDrop 1,000 spectrophotometer (Thermo Fisher Scientific, Waltham, USA). Then, cDNA was synthesized according to the protocol of the PrimeScriptTM II 1st Strand cDNA Synthesis Kit (Takara, Tokyo, Japan). Quantitative real-time PCR was performed using a CFX Connect Detection System (Bio-Rad, Hercules, CA, USA). Primers used in this study are listed in Supplementary Table S2.

### 2.10. Microbial composition analysis

In short, the V3-V4 regions of the 16S rRNA genes were amplified using the forward primer 341F(5′-CCTAYGGGRBGCASCAG-3′) and the reverse primer 806R(5′-GGACTACHVGGGTWTCTAAT-3′). PCR products were recovered and purified by using GeneJET Extraction Kit (Thermo Fisher Scientific, Wilmington, USA) after gel electrophoresis. Validated libraries were sequenced on the IonS5TMXL platform provided by Personalbio (Shanghai, China). The 16S rRNA gene sequence data were deposited in the National Center for Biotechnology Information (NCBI) Sequence Read Archive (SRA) under the accession number PRJNA877369.

### 2.11. Statistical analyses

Data obtained were analyzed by two-factor ANOVA and Duncan multiple comparison method (SPSS 22.0; IBM-SPSS Inc., Chicago, IL, USA). The results were expressed as mean and standard error (SEM), and *p* < 0.05 was the significant difference level.

## 3. Result

### 3.1. Growth performance

The body weight (BW), average daily gain (ADG), average daily feed intake (ADFI), feed intake: body gain (F: G), diarrhea rate, and diarrhea index are shown in Table 1. There was no significant effect of ZnO or CT supplementation on BW, ADG, ADFI, and F/G (*p* > 0.05). Dietary supplementation with ZnO or CT decreased the diarrhea rate and diarrhea index from 0 to 14 days, 15 to 28 days, and 0 to 28 days, compared with CON group (*p* < 0.05). And compared to CON group, ZnO + CT group also decreased the diarrhea rate and diarrhea index from 0 to 14 days, 15 to 28 days, and 0 to 28 days (*p* < 0.05), but it is not significant compared with ZnO group (*p* > 0.05).

**Table 1:** Effects of ZnO and CT on growth performance of weaned piglets in K88-challenged environment.

	CON	ZnO	CT	ZnO + CT	SEM	*p*-value
BW, kg						
0 day	8.42	8.42	8.36	8.38	0.02	0.731
14 days	11.76	12.58	11.77	12.18	0.14	0.129
28 days	20.60	22.45	20.83	21.48	0.41	0.396
ADG, g						
0–14 days	238.77	297.30	243.37	270.87	10.28	0.152
15–28 days	631.55	704.52	647.14	664.64	23.57	0.747
0–28 days	435.18	500.83	445.24	467.80	14.56	0.413
ADFI, g						
0–14 days	341.98	404.05	338.79	347.74	11.42	0.135
15–28 days	852.32	1046.31	897.26	970.71	30.80	0.118
0–28 days	597.16	725.18	618.03	659.23	18.65	0.064
F:G						
0–14 days	1.44	1.39	1.41	1.29	0.03	0.417
15–28 days	1.35	1.5	1.42	1.47	0.04	0.529
0–28 days	1.37	1.47	1.4	1.42	0.03	0.731
Diarrhea rate, %						
0–14 days	40.80a	4.02b	15.37b	2.87b	3.70	<0.001
15–28 days	31.03a	1.73c	18.16b	1.73c	3.12	<0.001
0–28 days	35.92a	2.87c	16.76b	2.30c	3.25	<0.001
Diarrhea index						
0–14 days	0.76a	0.12c	0.32b	0.13c	0.06	<0.001
15–28 days	1.24a	0.04c	0.66b	0.10c	0.11	<0.001
0–28 days	1.00a	0.08c	0.49b	0.11c	0.08	<0.001

BW = body weight; ADG = average daily gain; ADFI = average daily feed intake; F: G = feed intake: body gain; SEM = standard error of the mean. ^a,b^Means lacking common superscript letter indicated significant differences (*p* < 0.05) within a row.

### 3.2. Effects of ZnO and CT on serum antioxidant capacity, immunoglobulins, and inflammatory cytokines in weaned piglets with K88 challenged

**Figure 1:**
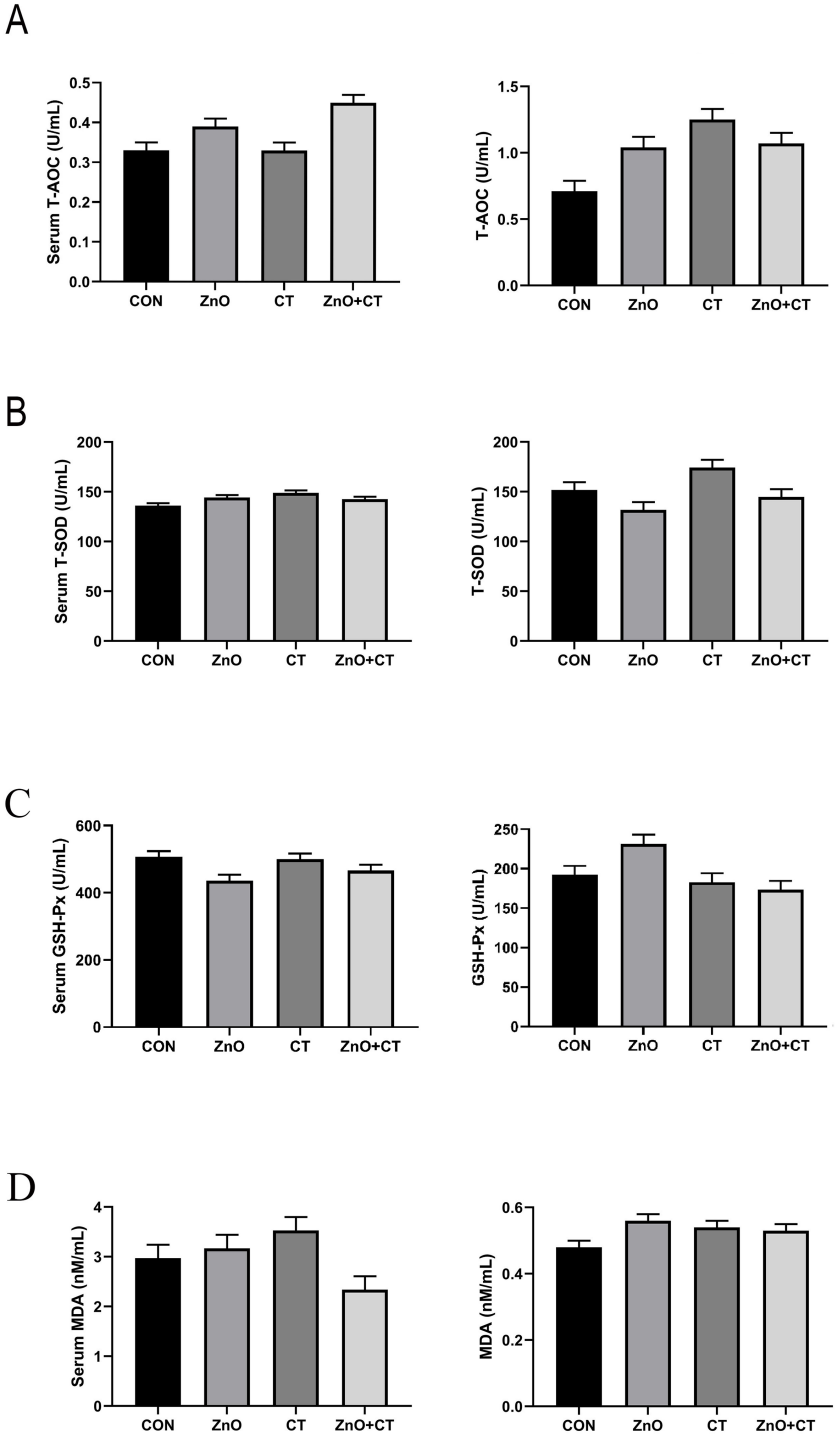
Effects of ZnO and CT on serum and liver antioxidant capacity of weaned piglets under K88-challenged environment. **(A)** The concentrations of T-AOC in serum and liver. **(B)** The concentrations of T-SOD in serum and liver. **(C)** The concentrations of GSH-Px in serum and liver. **(D)** The concentrations of T-MDA in serum and liver. T-AOC = total antioxidant capacity; T-SOD = total superoxide dismutase; GSH-Px = glutathione peroxidase; MDA = malondialdehyde. Values are means and standard errors represented by vertical bars (*n* = 6).

**Figure 2:**
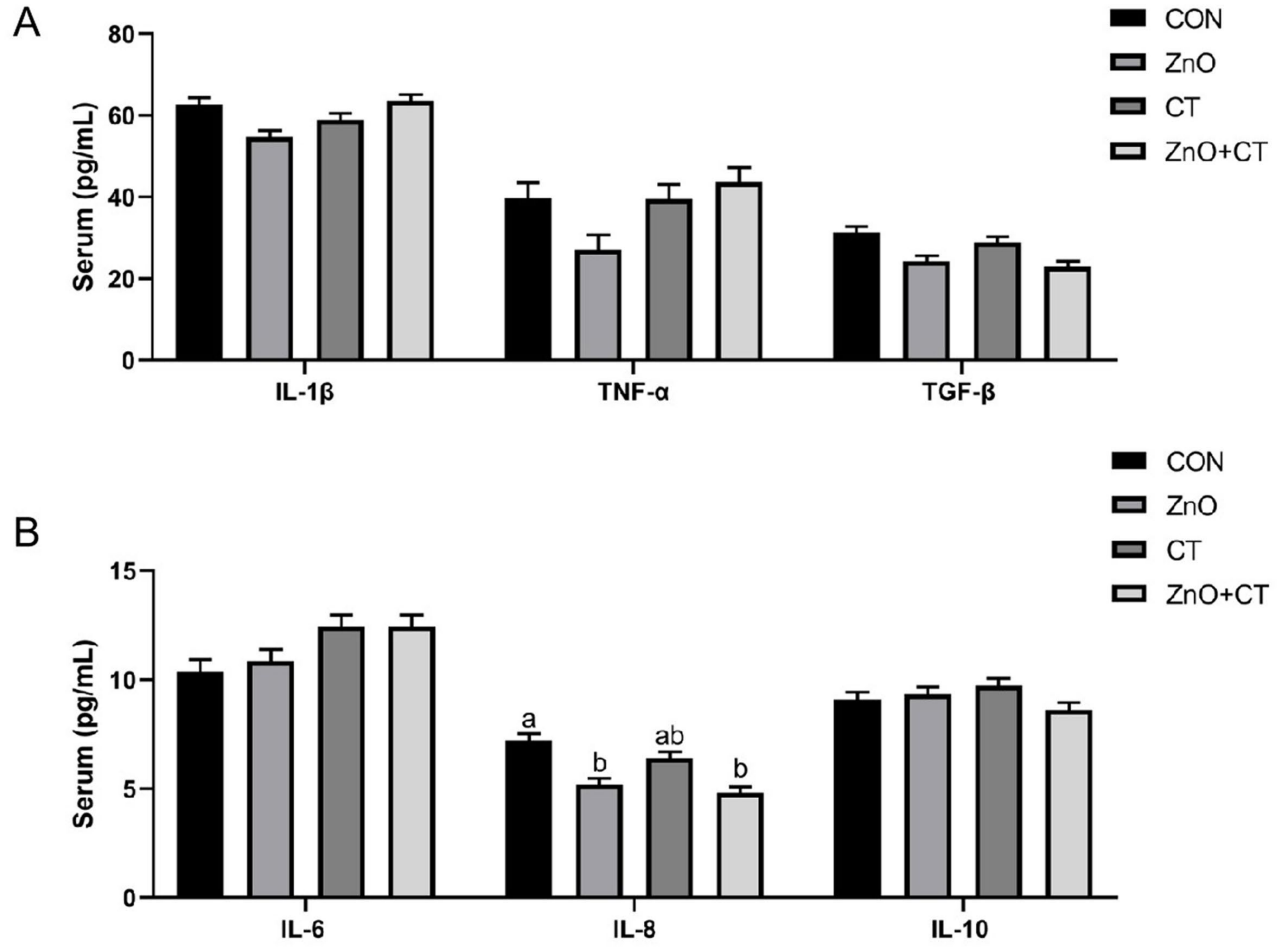
Effects of ZnO and CT on serum inflammatory factors in weaned piglets under K88-challenged environment. **(A)** The concentrations of IL-1β, TNF-α, and TGF-β. **(B)** The concentrations of IL-6, IL-8, and IL-10. IL-1β = interleukin-1β; IL-6 = interleukin-6; IL-8 = interleukin-8; IL-10 = interleukin-10; TNF-α = tumor necrosis factor; TGF-β = transforming growth factor-β. Values are means and standard errors represented by vertical bars (*n* = 6). ^a,b^Means lacking common superscript letter indicated significant differences (*p* < 0.05).

We observed the effect of ZnO or CT on the antioxidant capacity of piglets by detecting T-AOC, T-SOD, GSH-px, and MDA in serum and liver, and the results are shown in Figure 1. We found no significant effect of either ZnO or CT on the antioxidant capacity of piglets (*p* > 0.05). As shown in Table 2 and Figure 2, ZnO + CT group increased the concentration of immunoglobulin M (IgM), compared with CON and CT group (*p* < 0.05), but not significant compared with ZnO group (*p* > 0.05). And we found no significant effect of either ZnO or CT on the secretory immunoglobulin A (sIgA) of piglets (*p* > 0.05). In addition, the ZnO group and ZnO + CT group decreased the concentration of interleukin-8 (IL-8) compared to CON group (*p* < 0.05). However, serum inflammatory cytokines were not markedly affected by CT treatment.

**Table 2:** Effects of ZnO and CT on immunoglobulin contents in serum and intestinal mucosa of weaned piglets in K88-challenged environment.

	CON	ZnO	CT	ZnO + CT	SEM	*p*-value
Serum						
IgA, pg/ml	13.87	12.84	11.19	10.21	0.75	0.329
IgM, pg/ml	11.34bc	15.80ab	10.31c	16.27a	0.77	0.002
IgG, μg/ml	59.78	48.22	58.99	47.10	3.48	0.438
Intestinal mucosa						
Jejunum sIgA, ng/mg prot	2.90	3.88	3.14	3.59	0.15	0.081
Iluem sIgA, ng/mg prot	2.99	2.87	3.07	2.65	0.14	0.770

IgA = immunoglobulin A; IgM = immunoglobulin M; IgG = immunoglobulin G; sIgA = secretory immunoglobulin A; SEM = standard error of the mean. ^a,b^Means lacking common superscript letter indicated significant differences (*p* < 0.05) within a row.

### 3.3. The effects of ZnO and CT on intestinal barrier function

**Figure 3:**
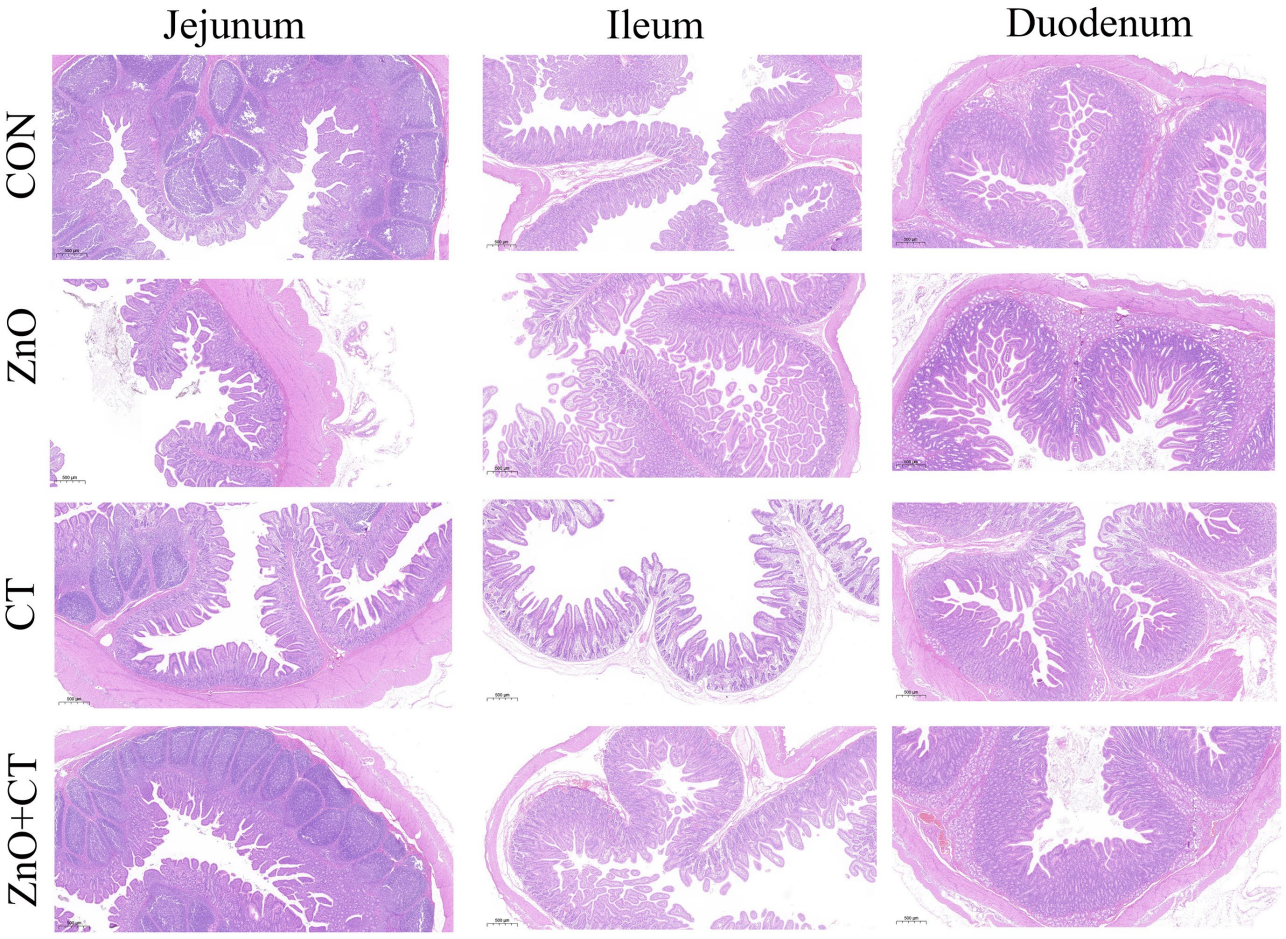
Effects of ZnO and CT on intestinal morphology of weaned piglets in K88-challenged environment.

**Figure 4:**
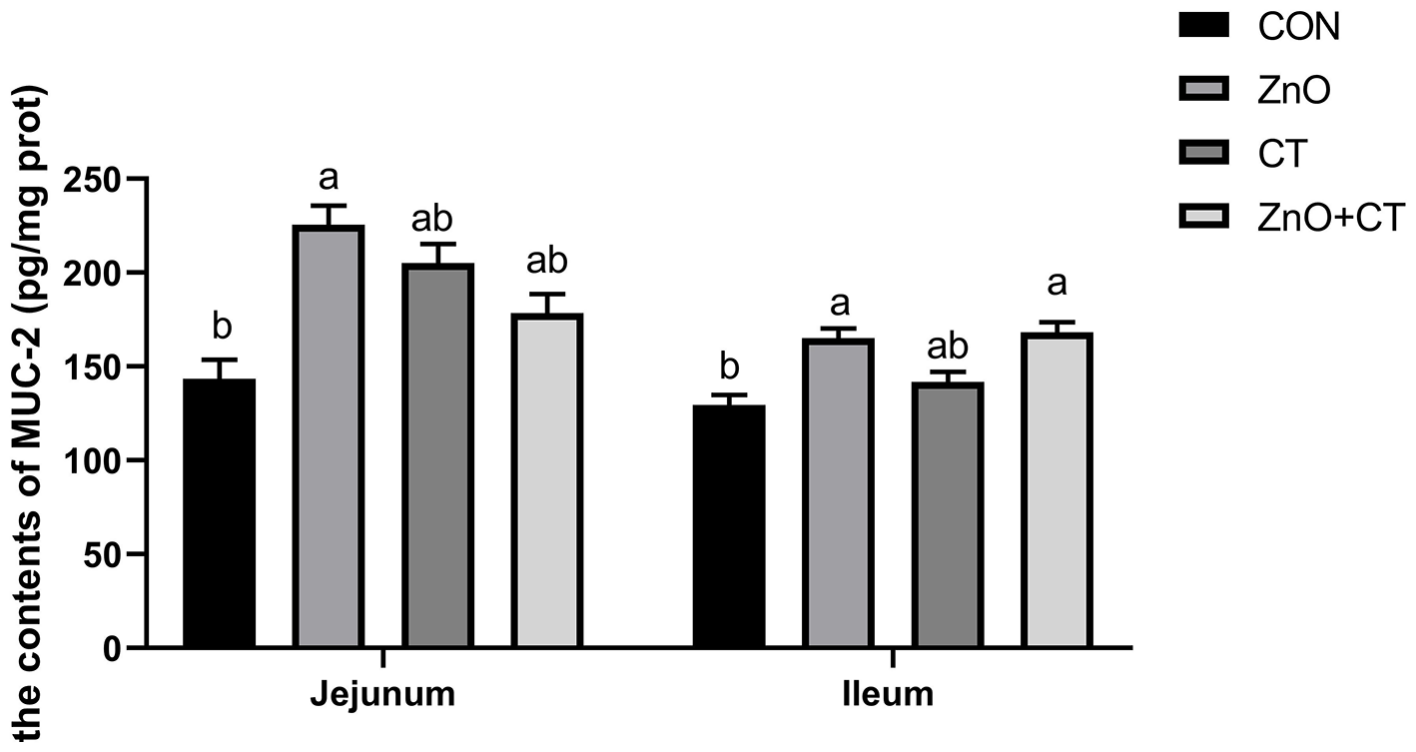
Effects of ZnO and CT on MUC-2 of weaned piglets in K88-challenged environment. MUC-2 = mucin-2. Values are means and standard errors represented by vertical bars (*n* = 6). ^a,b^Means lacking common superscript letter indicated significant differences (*p* < 0.05).

The intestinal morphology (duodenum, jejunum, and ileum) was observed by measuring the crypt depth, villus height, and the ratio of villus height to crypt depth (Figure 3 and Table 3). The morphology of intestinal villi was normal in ZnO groups and CT group, compared with the CON group. However, ZnO + CT group had a lower ileum villus height (*p* < 0.05), compared with the ZnO group. As shown in Figure 4, dietary supplementation of ZnO increased the MUC-2 content in jejunum and ileum mucosa (*p* < 0.05), compared with the CON group. And the ZnO + CT group also increased the MUC-2 content in ileum mucosa (*p* < 0.05), compared with the CON group. The effects of ZnO or CT on ZO-1 and Occludin gene expression in weaned piglets are shown in Table 4. We found ZnO or CT no significant effect of the mRNA expression of Occludin in the duodenum (*p* > 0.05). However, compared with the ZnO or CT group, the mRNA expression of Occludin in the duodenum was decreased in the ZnO + CT group (*p* < 0.05). And compared with the CON group, the mRNA expression of zonula occludens-1 (ZO-1) in the jejunum was increased in the ZnO or CT group and the ZnO + CT group (*p* < 0.05). In addition, compared with the CON group, the mRNA expression of Occludin in the ileum was increased in the ZnO or CT group (*p* < 0.05), but no significant change in the ZnO + CT group (*p* > 0.05).

**Table 3:** Effects of ZnO and CT on intestinal morphology of weaned piglets in K88-challenged environment.

	CON	ZnO	CT	ZnO + CT	SEM	*p*-value
Villus height, μm						
Duodenum	432.91	478.15	469.53	455.67	13.69	0.697
Jejunum	328.09	408.64	456.88	413.69	21.25	0.188
Ileum	284.54ab	367.25a	298.06ab	247.34b	14.68	0.020
Crypt depth, μm						
Duodenum	259.79	261.57	275.02	279.35	8.99	0.850
Jejunum	203.10	217.65	237.66	206.39	7.99	0.437
Ileum	184.04	182.31	198.57	175.26	6.05	0.610
VCR						
Duodenum	1.72	1.83	1.74	1.67	0.06	0.814
Jejunum	1.61	1.93	1.94	2.04	0.09	0.409
Ileum	1.58	2.05	1.56	1.43	0.09	0.096

SEM = standard error of the mean. ^a,b^Means lacking common superscript letter indicated significant differences (*p* < 0.05) within a row.

**Table 4:** Effects of ZnO and CT on intestinal tight junction gene expression in weaned piglets under K88-challenged environment.

	CON	ZnO	CT	ZnO + CT	SEM	*p*-value
Duodenum						
ZO-1	1.00	1.00	1.12	1.13	0.03	0.109
Occludin	1.00ab	1.05a	1.11a	0.87b	0.03	0.003
Jejunum						
ZO-1	1.01b	1.50a	1.66a	1.71a	0.07	<0.001
Occludin	1.03	0.85	1.01	1.02	0.04	0.394
Ileum						
ZO-1	1.06	1.43	1.31	1.40	0.07	0.293
Occludin	1.00b	1.27a	1.35a	1.13ab	0.04	0.003

ZO-1 = zonula occludens-1; SEM = standard error of the mean. ^a,b^Means lacking common superscript letter indicated significant differences (*p* < 0.05) within a row.

### 3.4. The effects of ZnO and CT on the gene expression of diarrhea-related channels In weaned piglets

Effects of ZnO or CT on diarrhea-related channel gene expression in weaned piglets under K88-challenged environment are shown in Table 5. We found that compared with the CON group, the mRNA expression of cystic fibrosis transmembrane conductance regulator (CFTR) in the jejunum and ileum was decreased in the ZnO or CT group and the ZnO + CT group (*p* < 0.05). In addition, the mRNA expression of the Na^+^/H^+^ exchanger (NHE3) in colon was lower in the ZnO or CT group and the ZnO + CT group than CON group (*p* < 0.05). And the mRNA expression of NHE3 in colon was lower in ZnO + CT group than ZnO group (*p* < 0.05).

**Table 5:** Effects of ZnO and CT on diarrhea-related channel gene expression in weaned piglets under K88-challenged environment.

	CON	ZnO	CT	ZnO + CT	SEM	*p*-value
Jejunum						
AQP3	1.01	1.00	1.10	1.08	0.04	0.731
AQP8	1.00	0.94	1.07	1.06	0.03	0.257
CFTR	1.02a	0.44b	0.53b	0.32b	0.06	<0.001
NHE3	1.02	0.99	0.97	1.02	0.03	0.951
SGLT1	1.01	0.86	0.87	0.87	0.03	0.126
NKCC1	1.00	1.06	1.03	1.03	0.03	0.958
Ileum						
AQP3	1.01	1.07	0.95	1.07	0.05	0.804
AQP8	1.04	0.82	0.94	0.86	0.05	0.366
CFTR	1.01a	0.58b	0.46b	0.42b	0.06	<0.001
NHE3	1.01	0.95	0.92	1.05	0.04	0.702
SGLT1	1.04	1.18	1.25	1.23	0.06	0.585
NKCC1	1.00	0.87	0.96	1.02	0.03	0.209
Colon						
AQP3	1.02b	1.28ab	1.40a	1.54a	0.06	0.007
AQP8	1.01	1.29	1.05	1.27	0.06	0.171
CFTR	1.05	0.76	0.94	1.01	0.05	0.145
NHE3	1.00a	0.49b	0.46b	0.27c	0.06	<0.001
SGLT1	1.03	1.08	1.02	1.02	0.04	0.966
NKCC1	1.01	0.99	0.97	0.87	0.04	0.692

AQP3 = Aquaporin3; AQP8 = Aquaporin8; CFTR = CFTR; NHE3 = Na+/H+ exchanger; SGLT1 = Na + −glucose cotransporter 1; NKCC1 = Na + -K + -2Cl- cotransport carrier 1; SEM = standard error of the mean. ^a,b^Means lacking common superscript letter indicated significant differences (*p* < 0.05) within a row.

### 3.5. The effects of ZnO and CT on colonic microflora of weaned piglets

**Figure 5:**
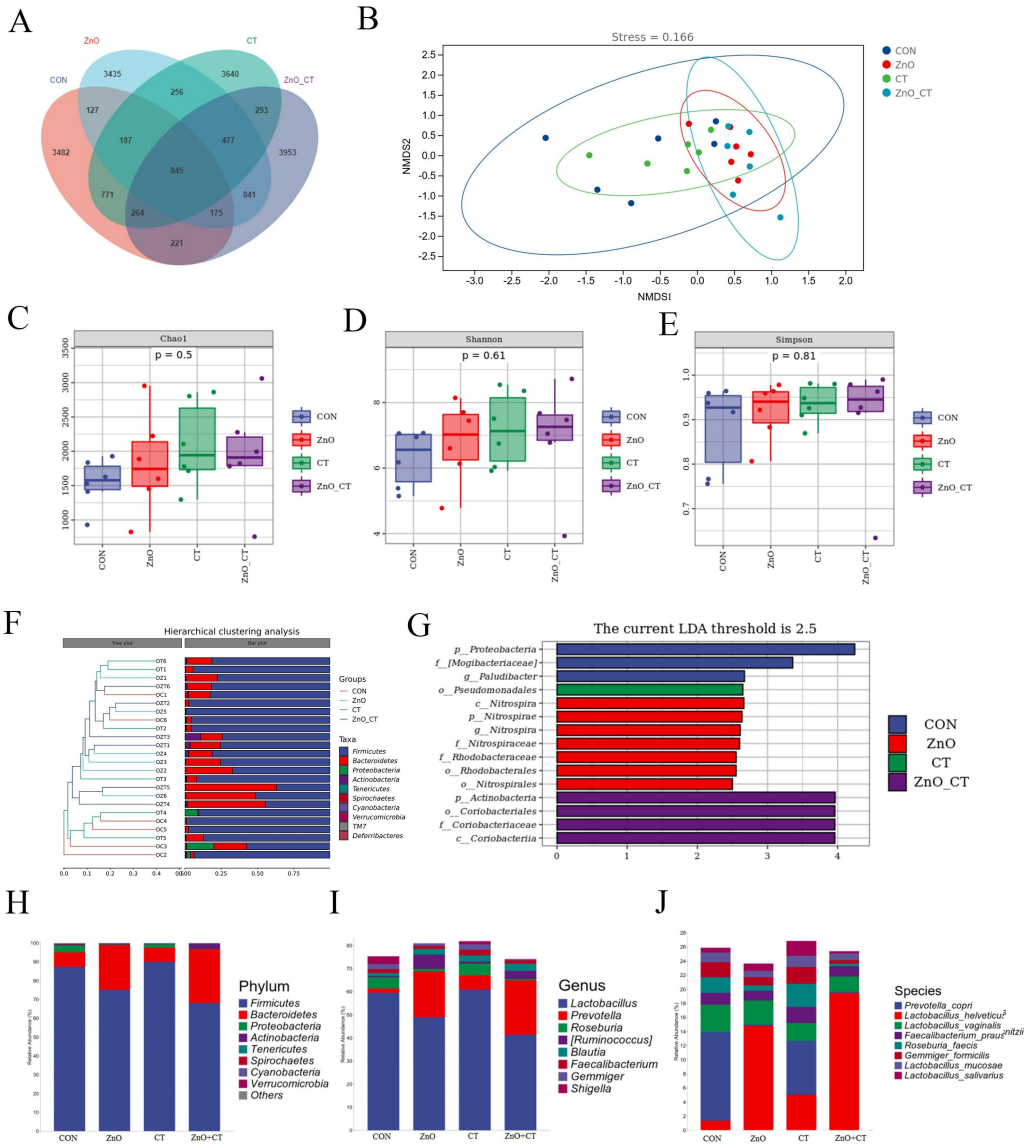
Effects of ZnO and CT on colonic microflora species composition of weaned piglets in K88-challenged environment. **(A)** Venn diagram illustrating common and special OTUs distributed among the four groups. **(B)** OUTs-based NMDS plot. **(C–E)** Species diversity and homogeneity were evaluated using Chao1, Shannon’s, and Simpson’s indices. **(F)** UPGMA clustering was conducted based on unweighted UniFrac distance. **(G)** Significantly different biomarkers in the four groups. **(H–J)** Effects of ZnO and CT on the composition of colonic microbiota in weaned pigs at the phylum, genus and species level.

Figure 5A shows the distributions of common and specific OTUs among the four groups. As shown in the NMDS plot (Figure 5B), we can see the clustering of the four groups of samples. We observed the Chao1, Shannon, and Simpson indexes in the all groups (Figures 5C–E), there was no significant difference (*p* > 0.05). A phenetic tree of the four groups was constructed based on unweighted UniFrac distance using UPGMA clustering method (Figure 5F). The LEfSe analysis identified discriminative species among the different groups (Figure 5G). Regarding the microbial composition at phylum level (Figure 5H), we observed that dietary supplementation of ZnO increased the relative abundance of *Bacteroidetes*, but decreased the relative abundance of *Firmicutes*. At the genus level (Figure 5I), the relative abundance of *Prevotella* was increased in ZnO group, compared with CON group, but the relative abundance of *Lactobacillus* was decreased. Furthermore, at the species level, dietary supplementation of ZnO increased the relative abundance of *Prevotella_copri*, but decreased the relative abundance of *Lactobacillus_helveticus* and *Lactobacillus_hamsteri* (Figure 5J). However, the microbial composition was not markedly affected by CT treatment.

## 4. Discussion

Pigs are one of the most important livestock and the most critical stage of their breeding is weaning. Post-weaning diarrhea (PWD) is a common disease in piglets after weaning, and its main pathogenic factor is enterotoxigenic *Escherichia coli* (Fairbrother et al., 2005). Dietary supplementation of traditional ZnO (2,000–4,000 mg/kg) can promote growth, relieve diarrhea, increase intestinal barrier function, and alter the composition of the intestinal microbiota in weaned piglets (Chen et al., 2015). However, it is still unknown whether 1,500 mg/kg ZnO or CT can improve piglet health as an alternative to traditional pharmacological doses of ZnO. In this study, we found that there was an upward trend in ADG an ADFI after ZnO or CT supplementation, but it was not significant, and F/G was also higher than the CON group. But we demonstrated that dietary supplementation of 1,500 mg/kg ZnO effectively inhibit diarrhea of weaned piglets in ETEC-polluted environment, which is consistent with previous studies (Yu et al., 2017; Wang et al., 2019). And supplementation of CT also inhibited diarrhea of weaned piglets in ETEC-polluted environment during the whole experimental period (from 0 to 28 days). However, the effect of ZnO in combination with CT to relieve diarrhea was similar to the effect of ZnO alone. Girard et al. found that gavage of weaned piglets with ETEC bacterial solution followed by dietary supplementation of 1% chestnut hydrolyzed tannins reduced the severity and duration of diarrhea, but did not completely prevent the occurrence of diarrhea (Girard et al., 2018), and the CT used in this study showed a similar function. The results indicated that 1,500 mg/kg ZnO or CT can reduce the risk of environmental pollution-induced diarrhea in piglets, but CT cannot be used as a substitute for ZnO.

Oxidative stress has negative effects on the health of weaned piglets. Usually, the body will produce SOD, CAT, GSH-Px, and other antioxidant enzymes to maintain balance in the body. If the body’s antioxidant capacity is insufficient, oxidative damage will occur to tissues and organs (Panov and Dikalov, 2020). But in this study, supplementation with ZnO or CT did not significantly affect T-AOC, T-SOD, GSH-px, and MDA in serum and liver.

Immunoglobulins are one of the important components of the body’s immune system and an important basis for humoral immunity. The increase in immunoglobulin concentration in pigs can improve immune function and promote the development of their own immune system, thus alleviating weaning stress (Macpherson et al., 2012). IgM is the first antibody produced when stimulated by an antigen (Teng et al., 2020). In this study, serum IgM were increased in ZnO + CT group, compared with CON group and CT group. Interestingly, there was insignificant difference in CT group. Therefore, we hypothesize that this result is due to the addition of ZnO, which can prevent improve immune status. To further explore the effects of ZnO and CT on intestinal immune function, we detected the levels of cytokine in serum. Interleukin-1β (IL-1β), IL-8, interleukin-6 (IL-6), and tumor necrosis factor (TNF-α) have a synergistic and inducible relationship and are responsible for regulating and mediating the body’s immune function (Wang et al., 2020; Wu et al., 2021). Some studies have shown that ETEC can increase the expression of these cytokines (Ren et al., 2020; Yi et al., 2021). In this research, dietary supplementation of ZnO reduced the concentration of IL-8 and had no significant effect on other cytokines. Wu et al. found that supplementation with ZnO (2,500 mg/kg) increased the mRNA expression levels of IL-8 in colon tissues, which is inconsistent with our results (Oh et al., 2021). In addition, ZnO + CT group also had a lower expression levels of IL-8 than CON group, but no significant than ZnO group. The above results indicated that supplementation with ZnO does not cause a rise in inflammatory factors and has some anti-inflammatory effects in ETEC-polluted environments. Our results indicated dietary supplementation of CT had no significant effect on cytokines. Persimmon-derived tannin was reported to reduce the expression of IL-1β, TNF-α, IL-6 (Matsumura et al., 2017; Kitabatake et al., 2021), which is inconsistent with our results. The possible reasons are related to the animal species and the sources of the CT.

Intestinal morphology is directly related to the digestion and absorption of nutrients and the mucosal barrier (Cera et al., 1988). In our study, dietary supplementation of ZnO or CT had no effect on intestinal morphology. But the villi height of the ileum was decreased in ZnO + CT group, compared with ZnO group. In addition, previous studies have shown that coated ZnO can improve ileum villus height (Sun et al., 2022). This may be due to the interaction between ZnO and CT. Liu et al. found that hydrolyzed tannin increased the villus height of ileum, which was inconsistent with our results. This may be caused by different forms of tannin (Liu et al., 2020). The intestinal barrier includes chemical barrier, mechanical barrier, and immune barrier. And mucin-2 is an important component of the mucus layer as the first barrier of the intestine (Yao et al., 2021). We further examined the levels of mucin-2 in ileum and jejunum, and the expression of tight junction genes in duodenum, jejunum, and ileum. Our results revealed that the content of muc-2 in jejunum and ileum mucosa was increased in ZnO group. And the content of muc-2 in jejunum mucosa increased in ZnO + CT group than CON group, but no significant difference compared to ZnO group. This indicated that CT could not significantly effect muc-2. In addition, dietary supplementation of ZnO combined with CT decreased the mRNA expression of Occludin in duodenum. But the mRNA expression of ZO-1 in jejunum was elevated in all treatment groups In addition, supplementation with ZnO or CT increased the mRNA expression of Occludin in ileum. Dietary supplementation with conventional doses of ZnO increased the mRNA expressions of ZO-1 in jejunum (Xia et al., 2017). Moreover, due to the complexity of the intestinal environment of piglets, the effects of ZnO or CT on each intestine may not be consistent. As our experimental results shown, supplementation of ZnO or CT increased the expression of Occludin in duodenum and ileum, but does not affect expression of Occludin in duodenum. The above results suggest that ZnO (1,500 mg/kg) and CT may have a potential function that can parallel the doses of traditional ZnO in improving the intestinal barrier of weaned piglets.

Diarrhea occurs mainly due to abnormal transport of electrolytes and water. Numerous transporter proteins related to electrolyte absorption are present in intestinal epithelial cells, such as NHE3, Na + −glucose cotransporter 1 (SGLT1), and water channel protein (AQP). In addition, the transporter proteins related to secretion are mainly CFTR and Na + -K + -2Cl- cotransport carrier 1 (NKCC1; Singh et al., 2014). To further explore the potential mechanism of ZnO and CT reducing diarrhea, we detected diarrhea-associated transporter protein genes by RT-PCR. AQP3 and AQP8 are closely associated with water transport in the colon (Zhao et al., 2016). AQP3 and AQP8 have been reported to be down-regulated in rats with colitis and ETEC induced diarrhea (Zhao et al., 2014; Yue et al., 2020). In our research, we found that the expression of AQP3 were up-regulated in CT group compared with the CON group, suggesting that CT prevent diarrhea by promoting water reabsorption and reducing the water content of stool. And there was an up-regulated trend of AOP3 in ZnO group. NHE3 plays an important role in the absorption of Na + in the intestinal lumen, and NHE3 knockout mice developed chronic diarrhea (Gawenis et al., 2002). And studies have shown that ETEC produces heat-stable enterotoxin or heat-labile enterotoxin which recognizes specific receptors and turns on CFTR, ultimately leading to watery diarrhea (Ren et al., 2022). In our study, dietary supplementation of ZnO or CT decreased the mRNA expression of CFTR in jejunum and ileum. In addition, the mRNA expression of NHE3 was down-regulated in ZnO or CT group. And the expression of NHE3 in ZnO + CT group was lower than that in ZnO group. Diarrhea leads to inhibition of NHE3 expression, which means that the intestinal absorption of Na + is reduced (Niu et al., 2021). Interestingly, the reduced expression of NHE3 in this study did not cause diarrhea in piglets, so we speculate that it may be that AQP in the colon has a stronger ability to interfere with water reabsorption than NHE3. The above results suggested that the potential mechanism of ZnO or CT anti-diarrhea is to reduce the expression of CFTR in the small intestine to prevent it over-activation and decrease intestinal fluid secretion. Moreover, CT can enhance the expression of AOP3 in the colon to promote water reabsorption. These results implied that the mechanism of CT to inhibit diarrhea was similar to that of ZnO.

Gut microbes are an important part of the animal organism and have a significant impact on animal health (Valdes et al., 2018). At the phylum level, the microbiota can be divided into three dominant phyla: *Bacteroides*, *Firmicutes*, and *Actinomycetes*. The ratio of *Firmicutes* to *Bacteroides* is an important indicator to evaluate the balance of the intestinal microbial community (Mariat et al., 2009). An increased *Firmicutes*/*Bacteroidetes* ratio has been observed in ZnO group. Ley et al. reported that an increase in *Firmicutes*/*Bacteroidetes* ratio was directly related to weight gain (Ley et al., 2006). *Lactobacillus* is considered to be a beneficial species in the intestinal flora. *Prevotella* is known to be one of the indispensable floras for the production of short-chain fatty acids (SCFAs), which metabolize a variety of complex oligosaccharides and polysaccharides and also protect animals from intestinal inflammation (Megahed et al., 2019). In this study, the abundance of *Prevotella* increased in the ZnO group, while the abundance of *Lactobacillus* decreased. However, previous studies have found that ZnO reduces the abundance of harmful bacteria and also reduces the abundance of *Lactobacillus* (Pajarillo et al., 2021). The above results suggest that ZnO can improve growth performance by regulating the composition of intestinal flora.

## 5. Conclusion

In K88-challenged environment, ZnO could alleviate diarrhea, enhance intestinal function, and alter the composition of the intestinal flora in piglets. Similar to ZnO, dietary supplementation of CT also showed the potential to improve intestinal function and alleviate diarrhea. The mechanism by which CT alleviated diarrhea may have been to reduce intestinal fluid secretion by decreasing CFTR expression and promote water reabsorption by increasing AQP3 expression. Moreover, the application of zinc oxide in combination with CT did not show a good improvement.

## Data availability statement

The datasets presented in this study can be found in online repositories. The names of the repository/repositories and accession number(s) can be found at: https://www.ncbi.nlm.nih.gov/, PRJNA877369.

## Ethics statement

The animal study was reviewed and approved by the Animal Care and Use Committee of Guangdong Academy of Agricultural Sciences.

## Author contributions

HY: conceptualization, validation, formal analysis, investigation, visualization, and writing—original draft. ZW and BY: methodology, validation, and formal analysis. XY, QW, YQ, and SH: investigation and validation. KG and YX: investigation and visualization. LW: conceptualization, supervision, project administration, and resources. ZJ: conceptualization, supervision, project administration, and funding acquisition. All authors contributed to the article and approved the submitted version.

## Funding

This study was financially supported by Start-up Research Project of Maoming Laboratory (2021TDQD002), Key Projects of Maoming Laboratory (2022ZD003), the earmarked fund for China Agriculture Research System (CARS-35), special project for rural revitalization strategy in Guangdong Province (2023TS-3-1), and the Science and Technology Program of Guangdong Academy of Agricultural Sciences (R2020PY-JG009).

## Conflict of interest

The authors declare that the research was conducted in the absence of any commercial or financial relationships that could be construed as a potential conflict of interest.

## Publisher’s note

All claims expressed in this article are solely those of the authors and do not necessarily represent those of their affiliated organizations, or those of the publisher, the editors and the reviewers. Any product that may be evaluated in this article, or claim that may be made by its manufacturer, is not guaranteed or endorsed by the publisher.
