# Supplementary material for: Effects of zinc oxide and condensed tannins on the growth performance and intestinal health of weaned piglets in ETEC-challenged environment
Source: Front Microbiol. 2023 Apr 27;14:1181519. doi: 10.3389/fmicb.2023.1181519 (PMC10172512; doi:10.3389/fmicb.2023.1181519)
Supplement: Supplementary file 1 [file Table_1.DOCX]

**Table S1. The formulations and chemical composition of the basal diet**

| Ingredient | Content, % | Energy and nutrient composition | Content, % |
| --- | --- | --- | --- |
| Corn, 8 % | 28.45 | NE, MJ/kg | 15.00 |
| Extruded corn, 8 % | 10.00 | CP | 21.50 |
| Soybean flour, 37 % | 14.00 | SID Lys, % | 1.44 |
| FSBM, 50 % | 11.50 | SID Met | 0.44 |
| bean pulp, 46 % | 7.00 | SID Thr | 0.85 |
| fish meal, 63 % | 3.00 | SID Try | 0.26 |
| LPWP, 3 % | 15.00 | SID Val | 0.92 |
| WPC, 76 % | 1.00 | SID Ile | 0.76 |
| Soybean oil | 1.00 | Ca | 0.81 |
| saccharose | 3.00 | Total P | 0.56 |
| NaHCO_3_ | 0.20 | STTD P | 0.42 |
| NaCl | 0.25 |  |  |
| CaHPO_4_ | 0.60 |  |  |
| Calcium citrate | 1.85 |  |  |
| *L*-Lys·HCl | 0.55 |  |  |
| *DL*-Met | 0.15 |  |  |
| *L-Thr* | 0.20 |  |  |
| *L*-Trp | 0.05 |  |  |
| *L*-Val | 0.10 |  |  |
| Choline Chloride | 0.20 |  |  |
| titanium dioxide | 0.40 |  |  |
| Premix^1^ | 1.50 |  |  |
| Total | 100.00 |  |  |

^1^ The premix provided following per kg of the diet: vitamin A, 15 500 IU; vitamin D, 3500 IU; vitamin E, 37.5 mg; vitamin K 6.25 mg；vitamin B_1_ 3.75 mg；vitamin B_2_ 12.5 mg；vitamin B_6_ 10 mg；vitamin B_12_ 50 μg；vitamin B_5_ 18.75 mg；Folic acid 1.25 mg；biotin 100 μg; Mn (MnSO_4_•H_2_O), 80 mg; Zn (Zinc methionine), 100 mg; Fe (ferrous fumarate); 160 mg; Cu (Cu methionine), 40 mg; Co (CoSO_4_•7H_2_O) 0.6 mg; and Se (Na_2_SeO_3_), 0.3 mg; I（Calcium iodate）0.8 mg. Except for measured values of the crude protein, calcium and total phosphorus, others are calculated values.

FSBM = [fermented soybean meal](javascript:;); LPWP = low protein whey powder; WPC = [whey protein concentrate](javascript:;); NE = net energy; CP = crude protein.

**Table S2. Primers for the real-time PCR analysis**

| **Gene** | **Sequence (5’-3’)** | **Size, bp** | **Accession number** |
| --- | --- | --- | --- |
| ZO-1 | F: AGCCCGAGGCGTGTTT | 147 | XM_013993251 |
|  | R: GGTGGGAGGATGCTGTTG |  |  |
| Occludin | F: GCACCCAGCAACGACAT | 144 | NM_001163647 |
|  | R: CATAGACAGAATCCGAATCAC |  |  |
| AQP3 | F: CAGGCTCGAAAAGCACCTTC | 292 | NM_001110172.1 |
|  | R: GCGGGGTCTTCTAGGAGGTA |  |  |
| AQP8 | F: CAGGCTCGAAAAGCACCTTC | 246 | NM_001112683.1 |
|  | R: GCGGGGTCTTCTAGGAGGTA |  |  |
| CFTR | F: GAAAAGGCCAGCATCTTCTCCA | 413 | NM_001104950.1 |
|  | R: CCAAAAATGGCTGGGTGCAG |  |  |
| NHE3 | F:AGCTGGAGATCATAGACCAGGTG | 305 | XM_021077062.1 |
|  | R:CGGTGAAGAAGATGACGATGAG |  |  |
| SGLT1 | F:TCCTGGTCGTCTCCCTCTTC | 177 | NM_001012297.1 |
|  | R:AATGGTCTCTTCTGGGGCTTC |  |  |
| NKCC1 | F:CAAGAAAAGGTGCTGTGTC | 101 | NM_001256528.1 |
|  | R:GTAAGGACGCTCTGATGATT |  |  |
| β-actin | F: CTGCGGCATCCACGAAACT | 380 | XM_003124280 |
|  | R: AGGGCCGTGATCTCCTTCTG |  |  |
